# Unravelling alternative splicing patterns in susceptible and resistant *Brassica napus* lines in response to *Xanthomonas campestris* infection

**DOI:** 10.1186/s12870-024-05728-8

**Published:** 2024-10-30

**Authors:** Li Yang, Lingli Yang, Chuanji Zhao, Zetao Bai, Meili Xie, Jie Liu, Xiaobo Cui, Klaas Bouwmeester, Shengyi Liu

**Affiliations:** 1 Key Laboratory of Biology and Genetic Improvement of Oil Crops, Ministry of Agriculture, Oil Crops Research Institute, Chinese Academy of Agricultural Sciences, Wuhan, Hubei 430062 China; 2 Biosystematics Group, Wageningen University & Research, Droevendaalsesteeg 1, Wageningen, 6708 PB The Netherlands; 3 Present Address: School of Pharmacy, Xi’an Jiaotong University, Xi’an, China; 4 Present Address: National Key Laboratory of Crop Genetic Improvement, College of Plant Science and Technology, Huazhong Agricultural University, Wuhan, China

**Keywords:** *Brassica napus*, Black rot resistance, *Xanthomonas campestris* pv. *campestris*, RNA-seq, Alternative splicing

## Abstract

**Background:**

Rapeseed (*Brassica napus* L.) is an important oil and industrial crop worldwide. Black rot caused by the bacterial pathogen *Xanthomonas campestris* pv. *campestris* (*Xcc*) is an infectious vascular disease that leads to considerable yield losses in rapeseed. Resistance improvement through genetic breeding is an effective and sustainable approach to control black rot disease in *B. napus*. However, the molecular mechanisms underlying *Brassica*-*Xcc* interactions are not yet fully understood, especially regarding the impact of post-transcriptional gene regulation via alternative splicing (AS).

**Results:**

In this study, we compared the AS landscapes of a susceptible parental line and two mutagenized *B. napus* lines with contrasting levels of black rot resistance. Different types of AS events were identified in these *B. napus* lines at three time points upon *Xcc* infection, among which intron retention was the most common AS type. A total of 1,932 genes was found to show differential AS patterns between different *B. napus* lines. Multiple defense-related differential alternative splicing (DAS) hub candidates were pinpointed through an isoform-based co-expression network analysis, including genes involved in pathogen recognition, defense signalling, transcriptional regulation, and oxidation reduction.

**Conclusion:**

This study provides new insights into the potential effects of post-transcriptional regulation on immune responses in *B. napus* towards *Xcc* attack. These findings could be beneficial for the genetic improvement of *B. napus* to achieve durable black rot resistance in the future.

**Supplementary Information:**

The online version contains supplementary material available at 10.1186/s12870-024-05728-8.

## Background

Alternative splicing (AS) is a crucial regulatory mechanism at post-transcriptional level, which greatly contributes to transcriptome diversity and proteome complexity [1]. It allows a pre-messenger RNA to produce multiple splicing variants by selecting different splicing sites [2]. AS is regarded as a universal phenomenon in multicellular eukaryotes [3]. For example, AS occurs in approximately 90% of the intron-containing genes in human [4], 60% in Arabidopsis [5], and 40% in maize and cotton [6, 7]. AS events are classified into five different types based on the location of the alternative splicing sites: alternate donor (AD), alternate acceptor (AA), alternate position (change in both AD and AA), exon skipping (ES), and intron retention (IR) [6]. A plethora of studies focusing on patterns of AS indicated that the proportion of different AS events varies widely across different species. IR is highly prevalent in plants, whereas it is not common in animals and yeast [8]. Transcripts generated by AS usually contain premature termination codons (PTCs). In most cases, these transcripts are rapidly eliminated via the nonsense-mediated mRNA decay (NMD) pathway [9]. NMD-insensitive transcripts can encode protein isoforms that may differ in structure, stability, function, localization, and other properties [2, 9]. An increasing number of studies have shown that AS can affect a wide range of biological processes, such as plant growth and development, flowering, circadian clock function, and plant response to biotic/abiotic stresses [10–12].

Rapeseed (*Brassica napus* L., 2n = 38, AACC), an important source of edible oil and industrial crop, has gained global attention. It is an allotetraploid species derived from *B. rapa* (2n = 20, AA) and *B. oleracea* (2n = 18, CC) around 7,500 years ago [13]. *Xanthomonas campestris* pv. *campestris *(*Xcc*), a vascular pathogenic bacterium, is the causal agent of black rot disease that has detrimental impact on crop productivity in Brassica crops, including *B. napus* [14]. This disease is typically characterized by V-shaped necrotic lesions on leaves and blackening of veins, which can result in considerable yield losses, especially when infection occurs at seedling stages [14, 15]. Current rapeseed cultivars are lacking insufficient resistance to black rot, and the use of agrochemicals is discouraged due to the pressing need for environmental protection. Enhancing black rot resistance in rapeseed through genetic breeding strategies is seen as a potent and sustainable alternative.

Dissecting the complexity of Brassica*-Xcc* interaction is rather critical for the breeding of disease-resistant Brassica plants. Previous studies on physiological indicators have shown that defensive metabolites—including glucosinolates, flavonoids, and phenolics—accumulate significantly in incompatible Brassica-*Xcc* interactions [16–19]. Furthermore, recent transcriptome analyses of Brassica crops with contrasting black rot resistance levels revealed the roles of genes involved in receptor-mediated immune signalling, ROS homeostasis, and the glucosinolate pathway in *Xcc* resistance [20, 21]. Next to gene transcriptional regulation, post-transcriptional mechanism AS has recently been recognized as an important gene regulation process in plants to resist against pathogen attack [22, 23]. Multiple genes associated with receptor-mediated immune signalling were reported to undergo AS in response to pathogen invasion to facilitate efficient immune responses [22]. For example, two *WRKY* transcription factor genes in rice, i.e. *OsWRKY62* and *OsWRKY76*, were found to encode isoform variants associated with immune responses against the blast fungus *Magnaporthe oryzae* and the leaf blight bacterium *Xanthomonas oryzae* [24]. In *B. napus*, Ma et al. (2019) investigated the AS landscape upon infection by the fungal pathogen *Leptosphaeria maculans* (anamorph *Phoma lingam*) and indicated the importance of several transcription factors in pathogen resistance [25]. Similar work by Ma et al. (2020) highlighted the role of several DAS genes in response to necrotrophic fungal pathogen *Sclerotinia sclerotiorum* [26]. However, the dynamics of AS patterns in *B. napus* in response to *Xcc* infection have not yet been thoroughly investigated.

In this study, we performed a comparative differential AS analysis with three *B. napus* lines showing contrasting levels of black rot resistance. Diverse AS events between different comparing datasets were systematically investigated. Regulatory networks of candidate AS genes potentially having an impact on *Xcc* resistance were pinpointed via an isoform-based co-expression analysis. This study enhanced our understanding of how *B. napus* responds to *Xcc* infection at the post-transcriptional level.

## Results

### A high proportion of new transcripts was found in *B. napus* against *Xcc* infection

We previously found that *B. napus* lines ZS9m*Xcc*R-1 and ZS9m*Xcc*S-1, two EMS mutants originating from *B. napus* cultivar ZS9, showed enhanced resistance and enhanced susceptibility against *Xcc* infection, respectively [27]. To understand the effect of the AS landscape on these *B. napus* lines with contrasting levels of black rot resistance, RNA-seq data of 27 leaf samples at 0, 5, and 8 days post inoculation (dpi) with *Xcc* were downloaded from the NCBI database. These datasets were then subjected to quality filtering and reads mapping, followed by transcript assembly and isoform quantification. We subsequently identified a total of 175,228 putative transcripts, among which 42.3% (74,188) were not annotated in the *B. napus* reference genome Darmor-*bzh* and thus considered as novel transcripts (Table S1). More than 52,000 transcripts were found per sample, representing up to 63% of the total transcripts (Table 1). These results indicate a strong overlap of the novel transcripts across different samples and suggest their potential vital roles in response to *Xcc* infection.

**Table 1:** Number of identified transcripts and AS events in each sample

Sample	Novel transcripts (n)	Novel transcripts (%)	A3SS (n)	A5SS (n)	ES (n)	IR (n)	AS sum
ZS9_0	55 382	63.8	3 214	1 599	576	8 943	14 332
ZS9_5	56 048	64.1	3 348	1 737	606	8 052	13 743
ZS9_8	53 354	63.3	3 014	1 487	552	6 816	11 869
ZS9m*Xcc*R-1_0	55 982	64.9	3 584	1 740	680	9 026	15 030
ZS9m*Xcc*R-1_5	57 655	65.1	3 941	1 970	668	9 816	16 396
ZS9m*Xcc*R-1_8	56 142	64.7	3 645	1 802	669	8 333	14 449
ZS9m*Xcc*S-1_0	57 188	64.6	3 789	1 696	641	8 662	14 787
ZS9m*Xcc*S-1_5	52 951	63.0	3 069	1 415	542	6 575	11 601
ZS9m*Xcc*S-1_8	55 363	64.3	3 314	1 575	581	7 450	12 919

*Abbreviations: A3SS* Alternative 3’ splice site, *A5SS* Alternative 5’ splice site, *ES* Exon skipping, *IR* Intron retention

### AS landscapes in *B. napus* in response to *Xcc* infection

**Fig. 1:**
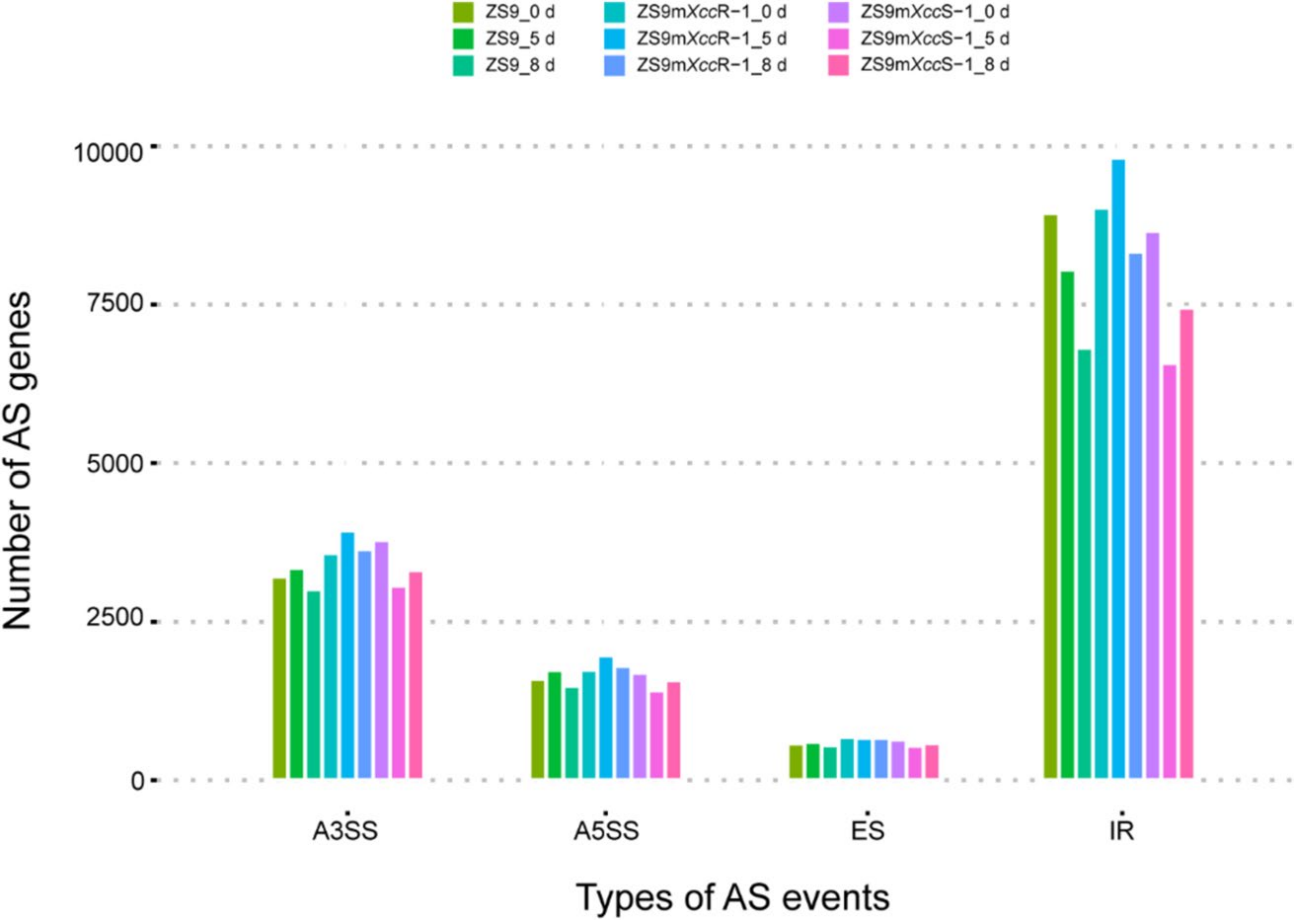
Alternative splicing (AS) landscapes of *B. napus* ZS9 and the EMS-lines ZS9m*Xcc*R-1 and ZS9m*Xcc*S-1 at 0, 5, and 8 dpi with *Xcc*. Abbreviations of the different types of AS events: A3SS, alternative 3’ splice site; A5SS, alternative 5’ splice site; ES, exon skipping; IR, intron retention

The assembled transcript file was further used to identify AS patterns and landscapes in the *B. napus* with different levels of black rot resistance. The number of different AS events detected in each *B. napus* line at different time points were counted (Table 1 and Fig. 1). IR was found to be the most common AS event, accounting for more than half of the total AS events in each sample, followed by A3SS and A5SS events, which comprised approximately 25% and 12% respectively. ES was the least frequent AS event, representing only about 4% in all samples. The resistant mutant line ZS9m*Xcc*R-1 was most affected by AS events, and A3SS and ES types were largely increased compared to that of the parental line ZS9. However, no significant difference in overall AS landscapes was observed across the different *B. napus* lines (Table 1).

### Identification of DAS genes between ZS9 and two EMS mutant lines showing contrasting levels of black rot resistance

**Fig. 2:**
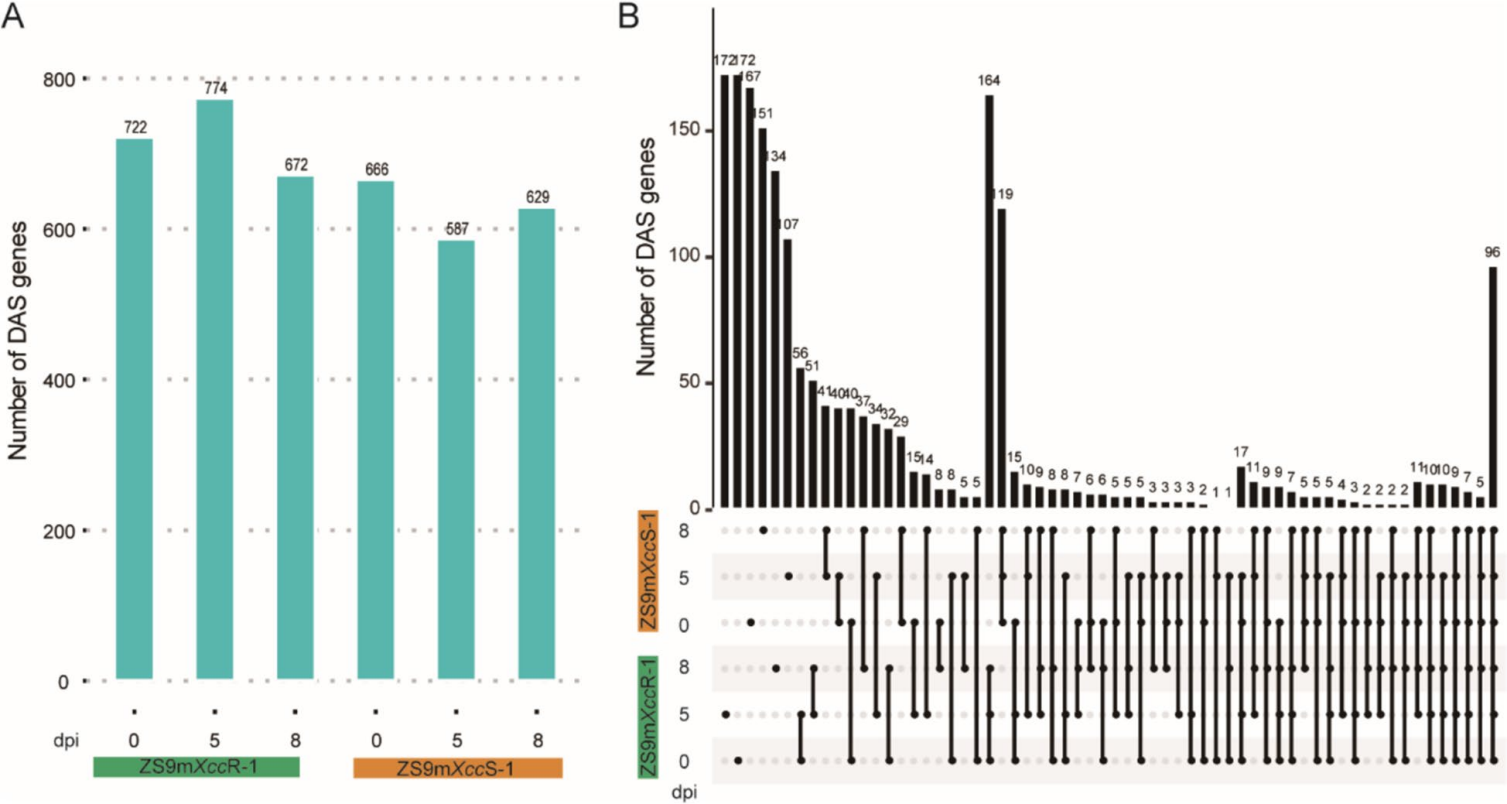
Comparative analysis of differential alternative splicing (DAS) genes in the EMS-lines ZS9m*Xcc*R-1 and ZS9m*Xcc*S-1 at different time points post *Xcc* infection. **A** Number of DAS genes in the resistant line ZS9m*Xcc*R-1 and the susceptible line ZS9m*Xcc*S-1 at 0, 5, and 8 dpi with *Xcc*. **B** Overlap of DAS genes in ZS9m*Xcc*R-1 and ZS9m*Xcc*S-1 at 0, 5, and 8 dpi with *Xcc*. The height of the bar chart indicates the number of DAS genes that are unique or shared in the corresponding group (the row on which the dots are located)

To study AS dynamics across *B. napus* lines that vary in black rot resistance, we compared the sorted bam file of the two EMS mutant lines with that of the parental line ZS9 at distinct timepoints upon *Xcc* inoculation. In total, 1,932 DAS genes were detected by integrating all comparative datasets (Table S2). A higher number of DAS genes was detected in the resistant line ZS9m*Xcc*R-1 at each time point after inoculation (Fig. 2A). During the time course of infection, the number of DAS genes was found to first increase at 5 dpi and then to decrease at 8 dpi in the resistant ZS9m*Xcc*R-1, whereas this distribution trend was exactly opposite in the susceptible line ZS9m*Xcc*S-1 (Fig. 2A). Comparative analysis of DAS genes between these two EMS lines revealed that approximately 25% (497) of the total DAS genes were identified at least at one time point in both mutagenized lines (Table S3). Among these, 96 genes exhibited differential alternative splicing across all comparative pairs (Fig. 2B). The number of DAS genes specifically detected in ZS9m*Xcc*R-1/ZS9 and ZS9m*Xcc*S-1/ZS9 were 781 and 654, accounting for a ratio of 40.4% and 33.9%, respectively (Table S3). These results suggested that the two EMS mutant lines with contrasting levels of black rot resistance have a quite distinct pattern of differential alternative splicing in response to *Xcc* infection.

### Functional annotation and enrichment analysis of DAS genes

The identified DAS genes were functionally annotated by performing BLAST similarity searches against the Arabidopsis protein database. In total, 1,682 of the 1,932 DAS genes were found to have homologs in *A. thaliana* (Table S2). One third of these homologous genes (630) were previously reported as AS genes according to the ASIP database [28], including those genes encoding RNA-binding proteins, cysteine proteins, calcium-dependent components, transcription factor, etc. (Table S2). All 1,932 DAS genes were subjected to Gene Ontology (GO) enrichment analysis using TBtools [29]. The results indicated that DAS genes uniquely present in the resistant line ZS9m*Xcc*R-1 were primarily associated with transmembrane transport activity, whereas those in the susceptible line were predominantly linked to mRNA processing. Most of the significantly enriched GO terms of DAS genes commonly found in these two lines were associated with protein phosphorylation and metabolic processes (Table S4).

### Co-expression network analysis identifies modules correlated with *Xcc* resistance

**Fig. 3:**
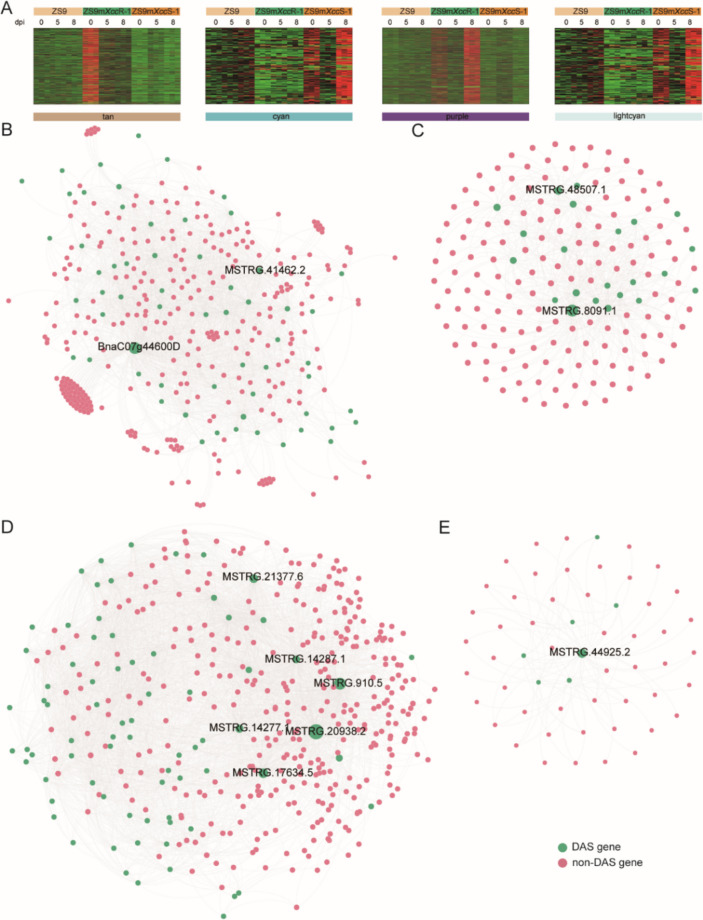
Isoform-based co-expression network analysis identifies modules that are highly correlated with the host–pathogen interactions. **A** Expression heatmaps of isoforms belonging to tan, cyan, purple, and lightcyan modules, respectively. **B**-**E** Weighted isoform co-expression networks of tan (**B**), cyan (**C**), purple (**D**), lightcyan (**E**) modules. Green and red nodes represent transcripts with DAS and non-DAS precursors, respectively. Node size refers to the number of connected edges within each module

To identify potential isoforms associated with plant immune responses to *Xcc* infection, a weighted co-expression network analysis (WGCNA) was conducted using 74,956 filtered transcripts with elevated expression levels. An isoform-based scale-free network was constructed using a soft threshold (β) of 9, resulting into the generation of 23 co-expression modules (Fig. S1). The number of isoforms/genes within each module varied largely, ranging from 56/54 in module darkgreen to 6679/5932 in module turquoise (Fig. S2). Combined with expression variations of isoforms within each module and the correlation analysis of module—treatment, we found that isoforms in the tan, cyan, purple, and lightcyan modules exhibited contrasting expression patterns across different *B. napus* lines and that they are closely associated with disease resistance or disease progression (ZS9m*Xcc*R-1_0, *r* = 0.92; ZS9m*Xcc*R-1_8, *r* = 0.75; ZS9m*Xcc*R-1_8, *r* = 0.88; ZS9m*Xcc*S-1_8, *r* = 0.76) (Fig. S1B). This result underscores the importance of further investigating the isoforms and genes within these modules. The expression patterns of the isoforms in tan, cyan, and purple modules were shown to be up-regulated at 0 or 8 dpi in the resistant line ZS9m*Xcc*R-1 and down-regulated in the susceptible line ZS9m*Xcc*S-1, and vice versa in the lightcyan module (Fig. 3A). We questioned whether these contrasting expression patterns among isoforms could elucidate the underlying mechanisms of plant immune resistance to *Xcc* infection. To answer this question, we first examined the enriched GO terms of the precursor genes within these modules. In the ZS9m*Xcc*R-1_0-related tan module, genes were mostly involved in protein phosphorylation, protein modification, and various metabolic processes (Fig. S3A). As infection time progressed, unique GO terms associated with genes in the ZS9m*Xcc*R-1_8-related cyan and purple modules were identified, including those involved in oxidation–reduction processes, response to stimuli, signal transduction, and receptor protein signalling (Fig. S3B, C). However, in the ZS9m*Xcc*S-1_8-related lightcyan module, the enriched GO terms were less directly related to host–pathogen interactions. Instead, they primarily encompassed various biosynthetic and metabolic processes, along with mRNA processing and splice site selection (Fig. S3D).

### Hub DAS genes potentially involved in *B. napus* resistance against* Xcc* infection

In a next step, we investigated how transcripts in each trait-related module were linked to commonly observed alternative splicing events. We first visualized the co-expression networks of transcripts in these modules, whose edges contain at least one transcript with a DAS precursor gene. The results showed that transcripts with DAS precursors were unevenly scattered in each network (Fig. 3B). Hereafter, we extracted the highly interconnected transcripts (|kME|> 0.8) with DAS precursors in each module (Table S5). The total number of DAS genes producing hub transcripts in the resistant line ZS9m*Xcc*R-1 and the susceptible line ZS9m*Xcc*S-1 were 39 and 35 across these four modules, respectively. This is slightly higher than the number of genes commonly found in both lines (29). Further functional annotation of these genes resulted in the identification of hub DAS isoforms/genes involved in black rot resistance, of which several are discussed below (Table S6).

#### Cell surface receptors and protein kinases

**Fig. 4:**
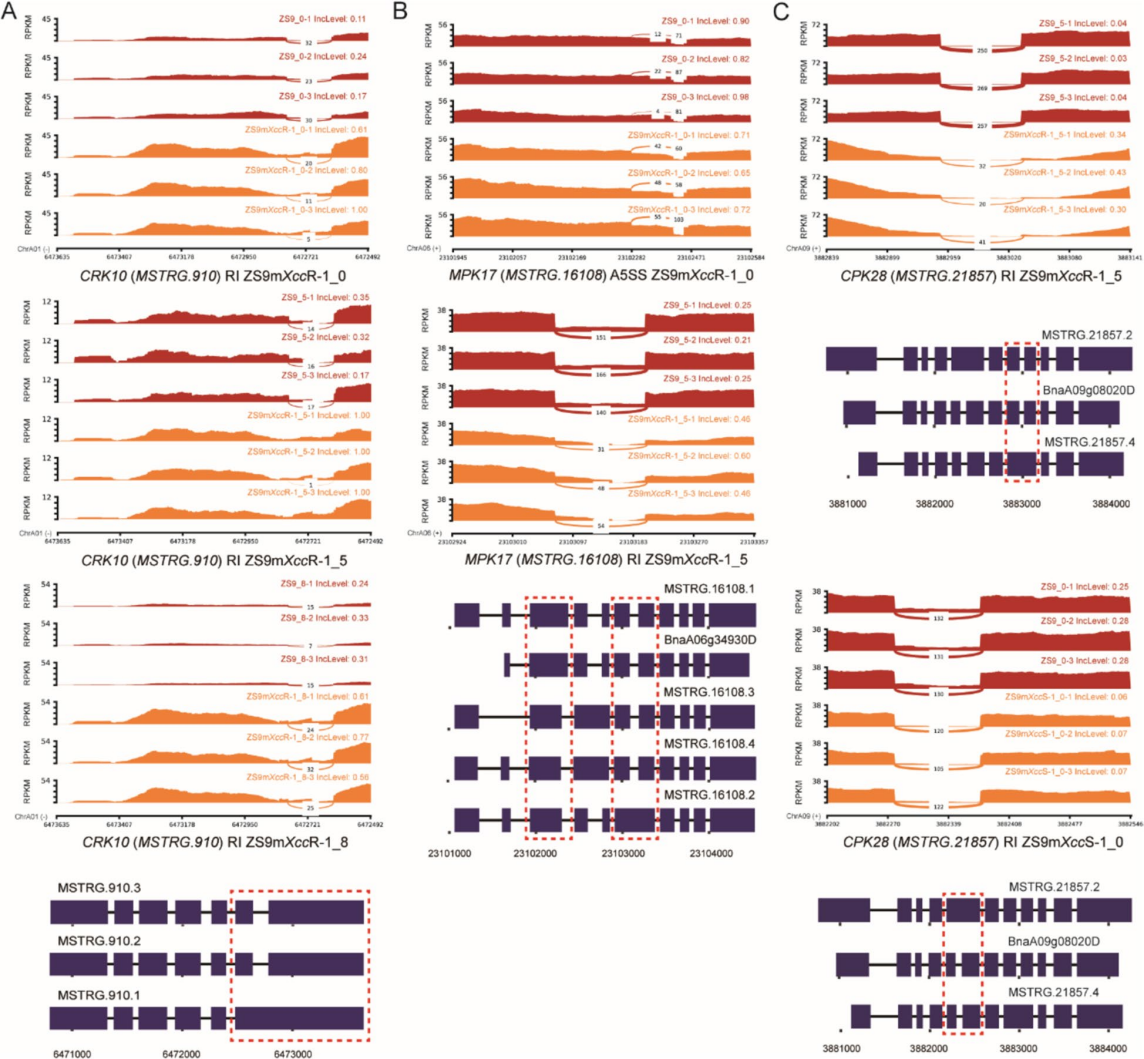
Sashimi plot and isoform structure of DAS genes related to immunity and defense signalling. **A** RI events of *CRK10* in the resistant line ZS9m*Xcc*R-1 at 0, 5, and 8 dpi with *Xcc*. **B** A5SS event of *MPK17* at 0 dpi and RI event at 5 dpi of resistant line ZS9m*Xcc*R-1 with *Xcc*. **C** RI events of *CPK28* in the resistant line ZS9m*Xcc*R-1 at 5 dpi and the susceptible line ZS9m*Xcc*S-1 at 0 dpi with *Xcc*. IncLevel values in Sashimi plots indicate the normalized proportion of AS events. Red dashed boxes across isoform structures represent the corresponding exon–intron region shown in the Sashimi plots. Blue rectangles and lines indicate exons and introns, respectively

**Fig. 5:**
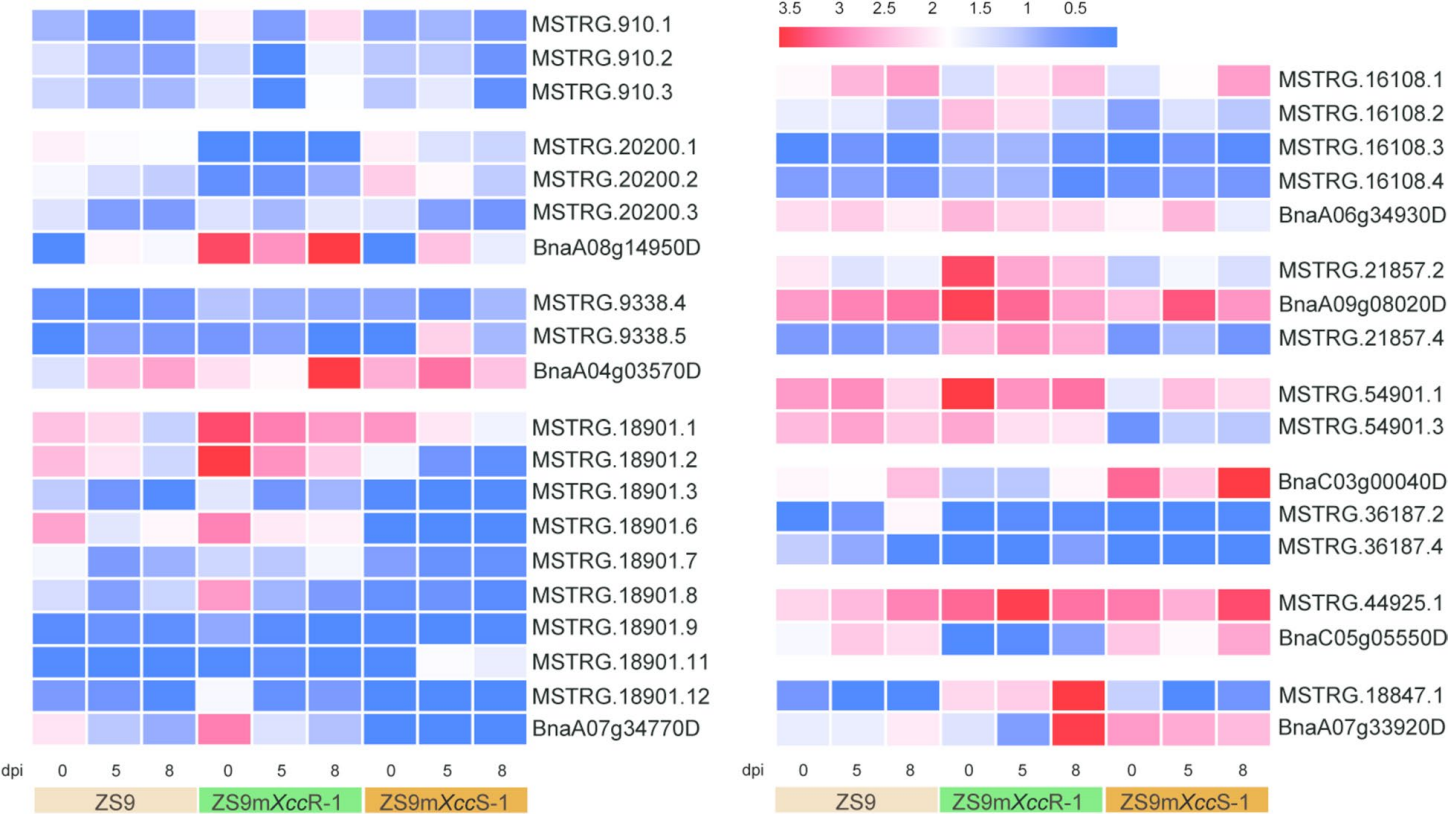
Expression heatmaps of each isoform of the candidate DAS genes involved in *B. napus*-*Xcc* interactions

Multiple copies of cysteine-rich receptor-like kinases (*CRKs*) with differential splicing patterns were identified in both resistant and susceptible *B. napus* lines (Table S6). For example, *CRK10* was found to undergo intron retention between isoform *MSTRG.910.3*/*MSTRG.910.2* and isoform *MSTRG.910.1* in *B. napus* lines at all three time points after *Xcc* infection (Fig. 4). The IncLevels of RI events increased significantly in the resistant line ZS9m*Xcc*R-1 compared to that in the parental line ZS9, indicating that *CRK10* was prone to have intron translation, which led to the formation of transcript *MSTRG.910.1*. This is also consistent with the difference in their expression levels, among which *MSTRG.910.1* was found to be largely up-regulated in the resistant line ZS9m*Xcc*R-1 (Fig. 5).

Besides DAS genes encoding receptor-like kinases (RLKs), a multitude of cytoplasmic RLCKs was found to display alternative splicing in different *B. napus* lines (Table S6). Among these, *PBL30* was shown to have different AS events in the resistant line ZS9m*Xcc*R-1 during the time course of *Xcc* infection, including A5SS, SE, and RI (Fig. S4). Detailed analyses of the Sashimi plots and transcript structures revealed that this gene tended to form transcript *BnaA08g14950D*, which was also found to be highly induced in expression in the resistant *B. napus* line ZS9m*Xcc*R-1 (Fig. 5). A related homolog *PBL1* was found to exhibit RI events in both *B. napus* lines with contrasting resistance levels against *Xcc* (Fig. S5). This potentially results into the intron-retained isoform *MSTRG.9338.4*, as suggested on the enhanced IncLevel values in both the resistant line ZS9m*Xcc*R-1 and the susceptible line ZS9m*Xcc*S-1 compared to ZS9. Moreover, the TIR-NBS-LRR resistance gene *MVA3.30*, which potentially plays a role in effector-triggered immunity, was identified to have various AS events in the compatible *B. napus*-*Xcc* interaction (Fig. S6A-E, Table S6). This led to more exon skipping events in the susceptible line ZS9m*Xcc*S-1, thus resulting in significantly reduced expression levels of most transcripts. The corresponding transcripts, however, were found to be largely induced in expression in the incompatible interaction (Fig. 5). This altered expression patterns of *MVA3.30* caused by differential splicing events indicates valuable clues into the functional roles of this disease resistance protein in the immune response following *Xcc* infection.

#### MAPK cascade and calcium signalling

Differential alternative splicing was also found in downstream regulatory genes associated with immune signalling. For example, one member of mitogen-activated protein kinase (MAPK) family, *MPK17*, was found to be alternatively spliced via A5SS and RI events in ZS9m*Xcc*R-1 at an early stage of infection (Fig. 4B). This leads to the generation of isoform *MSTRG.16108.2*, which also showed higher expression level in ZS9m*Xcc*R-1 at 0 and 5 dpi (Fig. 5). Alternative splicing closely associated with contrasting phenotypes was also observed in several genes involved in calcium signalling, such as *Calcium-dependent protein kinase 28* (*CPK28*), *Calmodulin-binding protein 60* (*CBP60*), and *Calreticulin 3* (*CRT3*) (Table S6). We found that *CPK28* exhibited intron retention across three transcripts (Fig. 4C). Among these, two transcripts (*MSTRG.21857.2* and *MSTRG.21857.4*) were significantly up-regulated in the resistant line ZS9m*Xcc*R-1 at all sampled time points post-inoculation. In contrast, *BnaA09g08020D* displayed a similar transcript level across the three analysed *B. napus* lines (Fig. 5).

#### Transcriptional regulation

**Fig. 6:**
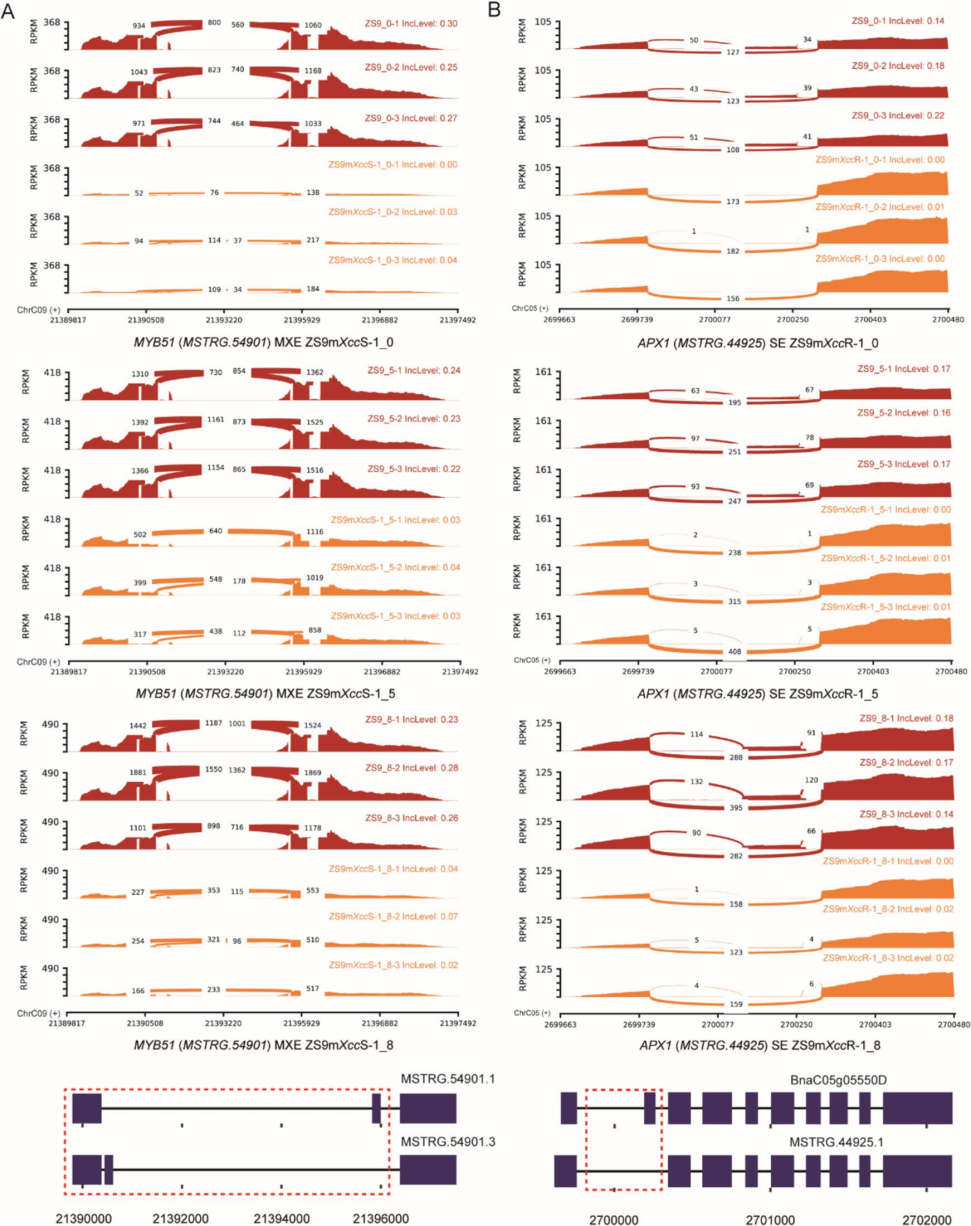
Sashimi plot and isoform structure of DAS genes related to transcriptional regulation and oxidation reduction. **A** MXE events of *MYB51* in the susceptible line ZS9m*Xcc*S-1 at 0, 5, and 8 dpi with *Xcc*. **B** SE events of *APX1* in the resistant line ZS9m*Xcc*R-1 at 0, 5, and 8 dpi with *Xcc*. IncLevel values in Sashimi plots indicate the normalized proportion of AS events. Red dashed boxes across isoform structures represent the corresponding exon–intron region shown in the Sashimi plots. Blue rectangles and lines indicate exons and introns, respectively

Several transcription factor genes (TFs) identified as hub DAS genes were found to play roles in immune signalling. Among these, *MYB51*—a member of the *R2R3-MYB* family—underwent differential splicing via mutually exclusive exon (MXE) events in the enhanced susceptible line ZS9m*Xcc*S-1 upon *Xcc* infection (Fig. 6A). The resultant isoform *MSTRG.54901.3* showed a relatively lower expression level in the compatible *B. napus*-*Xcc* interaction, while the other isoform had comparable expression in both incompatible and compatible interactions (Fig. 5). Furthermore, the Myb-related TF *LHY* that plays a role in circadian rhythm [30], was detected to show alternative splicing via an RI event in ZS9m*Xcc*S-1 at a later infection phase (Fig. S7A). Notably, the intron-retained isoform *BnaC03g00040D* was present in ZS9m*Xcc*S-1_8-related lightcyan module and its expression was found to be significantly up-regulated in the susceptible line ZS9m*Xcc*S-1 (Table S6, Fig. 5).

#### Oxidation reduction and other related processes

Two genes related to reactive oxygen species homeostasis were identified as DAS genes and shown to display differential splicing in the resistant line ZS9m*Xcc*R-1 (Table S6). An ascorbate peroxidase encoding gene (*APX1*) was found to undergo SE events between isoform *BnaC05g05550D* and *MSTRG.44925.1* at all time points after *Xcc* infection (Fig. 6B). The IncLevel of this gene was lower in ZS9m*Xcc*R-1 compared to the susceptible parental line ZS9, as well as the RPKM level of the second exon. This suggests that in the resistant line, *APX1* was prone to have ES events and to skip the second exon, resulting into isoform *MSTRG.44925.1*. Comparable work by Ma et al. (2020), who studied early AS patterns in *B. napus* upon *S. sclerotiorum* infection, also highlighted the importance of alternative splicing of *APX1* in pathogen resistance [26]. In other related biological processes, differential splicing in response to *Xcc* infection was found in a WD40 repeat-like protein encoding gene (Table S6), which is involved in multiple protein–protein interactions including protein scaffolding and subunit assembly [31]. Analysing Sashimi plots and transcript structures revealed that this gene tend to retain the fifth exon and produce isoform *MSTRG.18847.1* (Fig. S7B). This isoform showed a relatively high expression level in the resistant line immediately after *Xcc* inoculation, and much higher levels during the time course of infection (Fig. 5). Recently, this gene was also identified to have differential splicing in response to abiotic stresses, such as dehydration and cold [32].

## Discussion

Black rot caused by *Xcc* is one of the most devasting diseases in *B. napus*. Despite various transcriptome studies focusing on Brassica-*Xcc* interactions, our understanding of AS patterns in *B. napus* in response to black rot infection remained limited. To elucidate the role of AS in response to *Xcc* inoculation, we performed a comparative transcriptome analysis of three *B. napus* lines with contrasting levels of *Xcc* resistance at three time points post *Xcc* infection.

Different types of AS events in each *B. napus* line were identified, revealing that IR was the predominant event affecting alternative splicing, while ES was the least common (Fig. 1, Table 1). This frequency distribution of different AS events aligns partially with findings from previous studies on *B. napus* infected with *Leptosphaeria maculans* and *S. sclerotiorum* [25, 26], suggesting a common feature of AS in plants under biotic stress. Additionally, DAS genes present in different comparison datasets were determined. More DAS genes were observed in the incompatible interaction with line ZS9m*Xcc*R-1 at each time point following *Xcc* infection (Fig. 2A). This finding is consistent with the identification of a relatively higher number of differential expressed genes in this line, as reported in our previous study [27]. These results imply a stronger variation in gene expression levels and splicing patterns in the incompatible *Xcc*-Brassica interaction.

Co-expression network analysis at isoform level was performed to pinpoint defense-related hub DAS genes in this study. A large proportion of genes involved in pathogen recognition and downstream signalling was found to be differentially spliced in *B. napus* lines upon *Xcc* infection (Table S6). This includes genes encoding cell surface receptors and protein kinases functioning in immune responses. For example, various *CRKs*, reported to be involved in oxidative stress responses [33], were detected to have AS events in different *B. napus* lines (Table S6). A previous study reported that a frameshift mutation of *CRK10* in rice led to compromised immunity against *X. oryzae* pv. *oryzae* (*Xoo*) [34]. Multiple cytoplasmic RLCKs exhibiting AS were also identified, including several *PBL30* copies and other members of the large RLCK VII subfamily [35]. Here, we detected one copy of *PBL30,* i.e. *MSTRG.8626*, which corresponds to one of the hub genes (*BnaA03g53450D*) identified in our previous transcriptome study (Table S6) [27]. These results suggest that the regulatory protein PBL30 may play a central role in defense response against *Xcc* infection through both mechanisms of differential expression and alternative splicing. According to Sashimi plots and gene structure analysis, the related gene *PBL1* is more likely to transcribe into *MSTRG.9338.4* in both resistant and susceptible *B. napus* lines (Fig. S5), but no significant increase in its expression level was observed (Fig. 5). This might be masked by the elevated expression levels of *BnaA04g03570D*, an isoform that is highly expressed across all three *B. napus* lines.

Genes involved in downstream regulatory processes of immune signalling were also observed to have differential alternative splicing. *MPK17*, for example, was found to exhibit A5SS and RI events in the incompatible *B. napus*-*Xcc* interaction (Fig. 4B). MPK17 in Arabidopsis is known to affect peroxisome division during salt stress [36]. More recently, OsMPK17 was found to play a negative role in *Xa21*-mediated resistance to *Xoo* in rice [37]. Moreover, we also identified several DAS genes known to play crucial roles in immune homeostasis, including *CPK28*, *CBP60*, and *CRT3* [38–40]. One of the three produced isoforms by *CPK28* (i.e. *BnaA09g08020D*) did not show significant variation in expression levels across three different *B. napus* lines, while the other two isoforms were found to be highly expressed in the resistant line (Fig. 5). We speculate that this expression difference could be explained by the myriad roles of CPKs in plant immune signalling, including cell death regulation and promotion of ROS accumulation [41]. More recently, *CPK28* was found to undergo RI events upon activation of plant elicitor peptides, leading to the production of an intron-retained variant that encodes a truncated protein with reduced kinase activity. This in turn resulted in the diminished function of CPK28 as a negative regulator, thereby promoting stabilization of the cytoplasmic kinase BIK1 and subsequent immune defense [42].

TFs are proteins that bind to *cis*-regulatory specific sequences in the promoters of target genes to regulate their expression [43]. In this study, several TFs, including *MYB51*, were identified to be alternatively spliced in ZS9m*Xcc*S-1 (Fig. 6A). MYB51 is known to be an important regulator of indole glucosinolate biosynthesis [44]. An earlier study pointed out that glucosinolate metabolites are required for innate callose defense response in Arabidopsis [45]. *LHY*, another Myb-related TF was also found to undergo AS in the susceptible line (Fig. S7A). A recent study reported that *LHY*, along with *CCA1*, another MYB TF, plays a crucial role in nonhost resistance of Arabidopsis against the rice blast pathogen *Pyricularia oryzae* [46].

## Conclusion

In this study, we systematically explored and analysed the comparative transcriptome profiles of AS landscapes in *B. napus* lines exhibiting different levels of resistance to black rot. The aim was to identify crucial elements that show isoform variations in reaction to *Xcc* infection. A high proportion of novel transcripts was found in the studied *B. napus* lines (42.3%), of which 1,932 precursor genes were differentially spliced across the different datasets. By conducting an isoform-based co-expression network analysis, potential candidate genes playing a role in black rot resistance were pinpointed. Our results reveal that *B. napus* responds to *Xcc* infection by alternative splicing of diverse genes involved in various processes impacting immunity and stress signalling. This study provides valuable insights into the AS landscapes associated with black rot resistance in *B. napus* and may form a beneficial resource for molecular breeding of *Xcc-*resistant Brassica crops.

## Materials and methods

### RNA-seq data and transcriptome analysis

RNA-seq data of *B. napus* leaves before and after inoculation with *Xcc* were downloaded from the NCBI SRA database (BioProject number PRJNA748871) [27]. Raw data were firstly filtered by removing adapter sequences and low-quality reads using the NGSQC toolkit [47]. Generated clean reads were then mapped to the *B. napus* reference genome Darmor-*bzh* by Hisat2 v2.0.5 [13, 48]. Mapped reads were subsequently used to assemble putative transcripts, and the resultant general transfer format (GTF) file of each sample was merged into a non-redundant transcript dataset using StringTie v1.2.4 [49]. This dataset was used as a new reference for downstream expression analysis. Clean reads were re-mapped to quantify transcripts as Fragments Per Kilobase of exon model per million mapped reads (FPKM) [49]. A flow chart of the methodology and the code scripts used can be found in Fig. S8 and Data S1, respectively.

### AS landscape and DAS analysis

Astalavista v4.0 was used to analyze the different AS patterns present in each sample based on its assembled GTF file [50]. Alternative 3’ splice site (A3SS) is a junction that starts from one exon termination and ends inside the next exon. Alternative 5’ splice site (A5SS) is a junction that starts inside one exon and ends at the initiation of next exon. Exon skipping (ES) refers to a junction that starts from one exon termination and ends at the initiation of next exon. Intron retention (IR) refers to a junction that starts and ends within one exon. rMATS software was used to identify genes that underwent DAS events across different *B. napus* lines post *Xcc* infection by pairwise comparison of bam files [51]. DAS genes were defined with the thresholds of |ΔIncLevel|≥ 0.1 and FDR < 0.05. Sashimi plots were finally displayed to visualize significant DAS events by rmats2sashimiplot function (https://github.com/Xinglab/rmats2sashimiplot).

### Isoform co-expression network analysis

Weighted gene co-expression network analysis was performed with the top 50% transcripts using WGCNA package in R v3.5.1 [52]. Data clustering was conducted to examine the presence of outliers. Weighted co-expression network was constructed with a soft threshold power (β) of 9 when *R*^2^ > 0.85, followed by module identification and module-sample correlation analysis. Co-expression networks with edges containing at least one DAS gene and weighted values of co-expressed transcripts higher than 0.15 were kept and visualized in Gephi v0.9.7 [53]. Hub isoforms within each module were identified by in-house perl scripts with a threshold of |kME|> 0.8. Expression levels of significant isoforms were converted to log_2_(FPKM + 1) and used for heatmap visualization in R v3.5.1.

### Functional annotation and enrichment analysis

Functional annotation of *B. napus* DAS genes was implemented by BLAST similarity searches against the Arabidopsis protein database using an e-value threshold of 10^–5^. TBtools softwre was employed for GO enrichment analysis. GO terms with an adjusted *p*-value lower than 0.05 were designated to be significantly enriched [29].

## Supplementary Information

Supplementary Material 1: Figure S1. Topology analysis of co-expression networks. A, Scale independence and mean connectivity based on a set of soft thresholds. B, Heatmap showing correlations between modules and samples. Figure S2. Number of genes and transcripts within each module. Figure S3. Bubble diagrams showing the result of GO enriched terms in tan (A), cyan (B), purple (C), and lightcyan (D) modules. Bubble size indicates gene number. Figure S4. Sashimi plot and isoform structure of the DAS gene *PBL30*. A5SS (A), SE (B), and RI (C) events of *PBL30* in the resistant line ZS9m*Xcc*R-1 at 0, 5, and 8 dpi with *Xcc*. Figure S5. Sashimi plot and isoform structure of the DAS gene *PBL1*. RI events of *PBL1* in the resistant line ZS9m*Xcc*R-1 (A) and the susceptible line ZS9m*Xcc*S-1 (B) at 0, 5, and 8 dpi with *Xcc*. C, Isoform structure of *PBL1.* Figure S6. Various differential splicing events of the disease resistance gene *MVA3.30* in the susceptible line ZS9m*Xcc*S-1 during the time course of *Xcc* infection. A-D, SE events resulting into different isoforms. E, A5SS events resulting into different isoforms. Figure S7. Sashimi plot and isoform structure of the DAS genes *LHY* and *WD40-like*. A, RI events of *LHY* in the susceptible line ZS9m*Xcc*S-1 at 5 and 8 dpi with *Xcc*. B, SE events of *WD40-like* in the resistant line ZS9m*Xcc*R-1 at 0 and 5 dpi with *Xcc*. Figure S8. Flow chart for alternative splicing analysis. Table S1. FPKM values of all identified transcripts in 27 samples. Table S2. List of identified DAS genes. Table S3. DAS genes specifically or commonly present in ZS9m*Xcc*R-1 and/or ZS9m*Xcc*S-1 compared to ZS9. Table S4. Enriched GO terms of DAS genes specifically or commonly present in ZS9m*Xcc*R-1 and/or ZS9m*Xcc*S-1. Table S5. List of hub transcripts with DAS precursors in selected modules. Table S6. List of DAS hub genes potentially playing a role in black rot resistance. Data S1. Basic code scripts for each part of the analysis.

## Data Availability

Datasets supporting the findings of this study are available within the article and its supplementary materials. Raw data used in this study have been deposited in the NCBI SRA database under BioProject number PRJNA748871.

## Abbreviations

*Xcc*: *Xanthomonas campestris* pv. *campestris*
AS: Alternative splicing
AD: Alternate donor
AA: Alternate acceptor
ES: Exon skipping
IR: Intron retention
PTCs: Premature termination codons
NMD: Nonsense-mediated mRNA decay
DAS: Differential alternative splicing
WGCNA: Weighted gene co-expression network analysis
GO: Gene ontology
