# Supplementary material for: Unravelling alternative splicing patterns in susceptible and resistant Brassica napus lines in response to Xanthomonas campestris infection
Source: BMC Plant Biol. 2024 Oct 30;24:1027. doi: 10.1186/s12870-024-05728-8 (PMC11523580; doi:10.1186/s12870-024-05728-8)

Figure S7 Sashimi plot and isoform structure of the DAS genes *LHY* and *WD40-like*.
A, RI events of *LHY* in the susceptible line ZS9m*Xcc*S-1 at 5 and 8 dpi with *Xcc*.
B, SE events of *WD40-like* in the resistant line ZS9m*Xcc*R-1 at 0 and 5 dpi with *Xcc*.

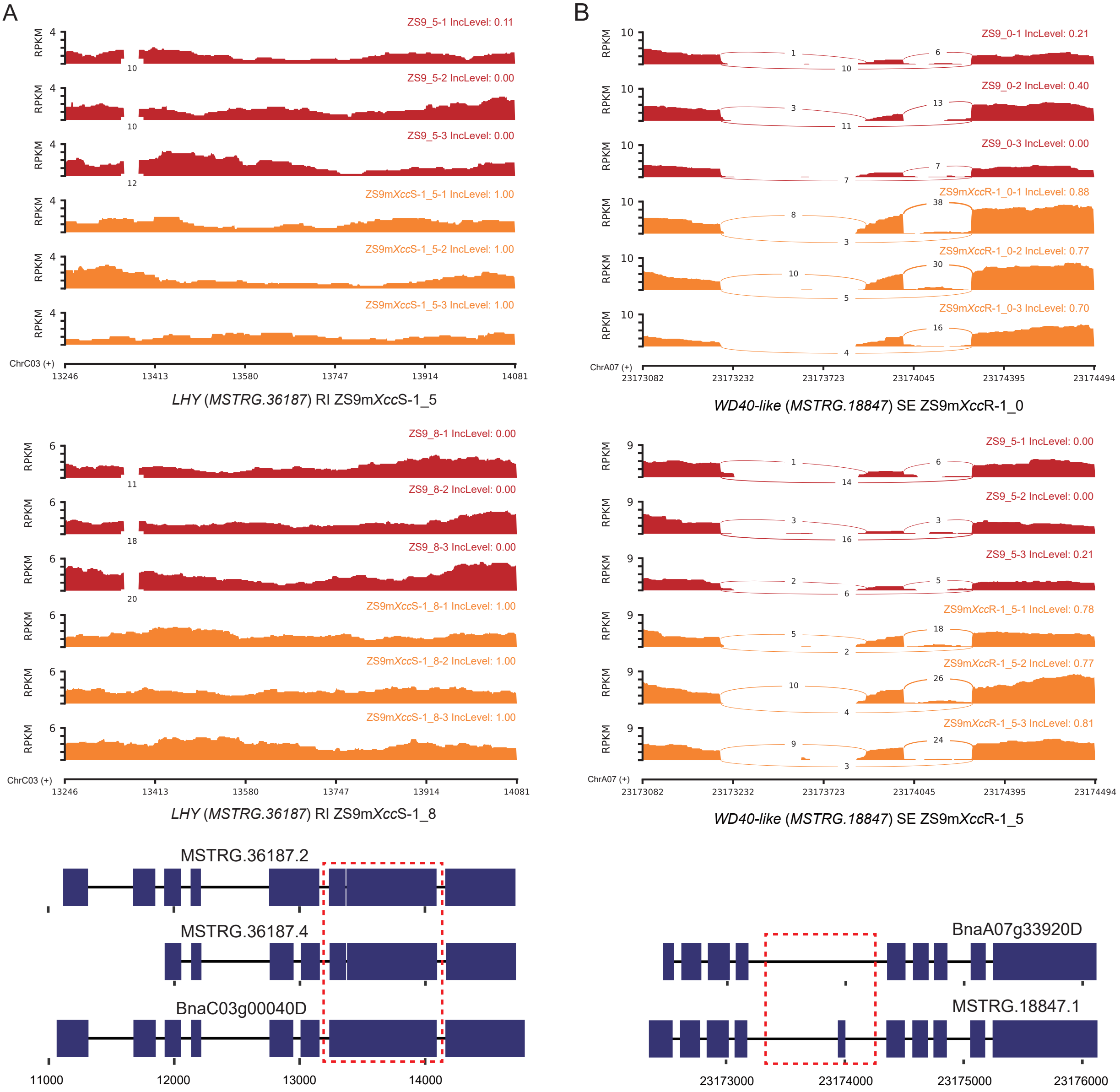

Supplement: Supplementary file 1 — Supplementary Material 1: Figure S1. Topology analysis of co-expression networks. A, Scale independence and mean connectivity based on a set of soft thresholds. B, Heatmap showing correlations between modules and samples. Figure S2. Number of genes and transcripts within each module. Figure S3. Bubble diagrams showing the result of GO enriched terms in tan (A), cyan (B), purple (C), and lightcyan (D) modules. Bubble size indicates gene number. Figure S4. Sashimi plot and isoform structure of the DAS gene PBL30. A5SS (A), SE (B), and RI (C) events of PBL30 in the resistant line ZS9mXccR-1 at 0, 5, and 8 dpi with Xcc. Figure S5. Sashimi plot and isoform structure of the DAS gene PBL1. RI events of PBL1 in the resistant line ZS9mXccR-1 (A) and the susceptible line ZS9mXccS-1 (B) at 0, 5, and 8 dpi with Xcc. C, Isoform structure of PBL1. Figure S6. Various differential splicing events of the disease resistance gene MVA3.30 in the susceptible line ZS9mXccS-1 during the time course of Xcc infection. A-D, SE events resulting into different isoforms. E, A5SS events resulting into different isoforms. Figure S7. Sashimi plot and isoform structure of the DAS genes LHY and WD40-like. A, RI events of LHY in the susceptible line ZS9mXccS-1 at 5 and 8 dpi with Xcc. B, SE events of WD40-like in the resistant line ZS9mXccR-1 at 0 and 5 dpi with Xcc. Figure S8. Flow chart for alternative splicing analysis. Table S1. FPKM values of all identified transcripts in 27 samples. Table S2. List of identified DAS genes. Table S3. DAS genes specifically or commonly present in ZS9mXccR-1 and/or ZS9mXccS-1 compared to ZS9. Table S4. Enriched GO terms of DAS genes specifically or commonly present in ZS9mXccR-1 and/or ZS9mXccS-1. Table S5. List of hub transcripts with DAS precursors in selected modules. Table S6. List of DAS hub genes potentially playing a role in black rot resistance. Data S1. Basic code scripts for each part of the analysis. [file 12870_2024_5728_MOESM1_ESM.zip › Fig.S7.pdf]
